# Melatonin Improves The Developmental Competence of Goat Oocytes 

**DOI:** 10.22074/ijfs.2018.5204

**Published:** 2018-03-18

**Authors:** Saghar Saeedabadi, Amir Hossein Abazari-Kia, Hoda Rajabi, Kazem Parivar, Mohammad Salehi

**Affiliations:** 1 Department of Biology, Faculty of Science, Islamic Azad University, Science and Research Branch, Tehran, Iran; 2 Department of Transgenic Animal Science, Stem Cell Technology Research Center, Tehran, Iran; 3 Cellular and Molecular Biology Research Center, Shahid Beheshti University of Medical Sciences, Tehran, Iran; 4 Department of Biotechnology, School of Advanced Technologies in Medicine, Shahid Beheshti University of Medical Sciences, Tehran, Iran

**Keywords:** Glutathione, Melatonin, Methylation

## Abstract

**Background:**

DNA methylation is one the epigenetic mechanisms, which is critically involved in gene expression. This phenomenon is mediated by DNA methyl-transferases and is affected by environmental stress, including in vitro maturation (IVM) of oocytes. Melatonin, as an antioxidant, may theoretically be involved in epigenetic regulation via reductions of reactive oxygen species. This study was performed to investigate DNA methylation and the possibility of goat oocyte development after treatment with different concentrations of melatonin.

**Materials and Methods:**

This experimental study was performed to investigate DNA methylation and the possibility of goat oocyte development after treatment with different concentrations of melatonin. For this purpose, oocytes with granulated cytoplasm were selected and co-cultured with at least two layers of cumulus cells in maturation medium with 10^-6^M, 10^-9^M, 10^-12^M and 0-M (as control group) of melatonin. Nucleus status, glutathione content and devel- opmental competence of the oocytes in each experimental group were assessed. Also, expression of genes associated with DNA methylation, including DNA methyltransferase 1 (DNMT1), DNA methyltransferase 3b (DNMT3b) and DNA methyltransferase 3a (DNMT3a) was evaluated by quantitative real time-polymerase chain reaction (RT-PCR).

**Results:**

According to our findings, the percentage of oocytes that reached the M-II stage significantly increased in the 10-12 M group (P<0.05). Also, a significant elevation of glutathione content was observed in melatonin-treated oocytes (P<0.05). Analysis of blastocyst formation revealed that developmental competence of the oocytes was higher than the control group (P<0.05). It was observed that melatonin treatment decreased expression levels of DNA meth- yltransferases (DNMTs) and global DNA methylation (P<0.05). In addition, the expression of melatonin receptor1A (MTNR1A) was detected in both cumulus and oocyte by RT-PCR.

**Conclusion:**

The results suggested that in goat model melatonin affects DNA methylation pattern, leading to an im- provement in the developmental competence of the oocytes.

## Introduction

Agood number of experiments have been designed to improve the in vitro production of goat embryos by adding numerous factors such as growth factors and antioxidants to maturation media (1, 2). 

Maturation of oocytes with a high level of competence is essential to obtain more high quality blastocysts (3). A study demonstrated that supplementation of MM with cysteine as an antioxidant enhances the level of intracellular glutathione (GSH) during in vitro maturation (IVM) and is maintained even after in vitro fertilization (IVF) (4). Another study suggested that addition of brain-derived neurotrophic factors as a growth factors increases GSH and improves developmental competence in ovine oocytes (5, 6). Nonetheless, the percentage of embryos that successfully develop into blastocysts is low (7).

During *in vitro* embryo production, various reacive oxygen species (ROS) scavengers such as l-ascorbic acid (vitamin c) and cysteine are used to protect oocytes and embryos from harmful effects of oxidative stress (OS) (8, 9). ROS has adverse effects on mitochondrial functions and epigenetic outcomes. OS strongly alters the expression of ten-eleven translocation (TET1), which is responsible for changing 5-methylcytosine to 5-hydroxymethylcytosine in bovine embryo (10).

Melatonin (N-acetyl-5-methoxytryptamine) is a potential antioxidant (11), which is produced from tryptophan and is secreted by the pineal gland. Melatonin is a well-known multifunctional molecule, as it mediates circadian rhythm, enhances immune-function, and regulates seasonal reproductive activity (12). It has been previously suggested that melatonin affects gene expression of several antioxidant enzymes such as glutathione peroxidase and superoxide dismutase (13). Melatonin can pass through cellular membrane and reach cytosol, inner mitochondria and nuclei, where it protects cells from signals that trigger apoptosis (6, 14). Several recent studies have shown that melatonin has beneficial effects on developmental competence of sheep, porcine, cattle, and mouse embryos, which is related to anti-oxidant capability of melatonin (15-20). 

According to a previous study, hydrolazine has an effect on methylation level by inhibition of methyltransferases. A review by Korkmaz et. al. (21) indicated that melatonin, like hydrolazine, can change methylation levels, which affects activation of genes without any changes in DNA sequencing.

Taken together, melatonin may have an effect on DNA methylation of goat oocytes as well as their developmental competence. Therefore, in this study the effects of different concentrations of melatonin on developmental competence, methylation dynamics and GSH level in goat oocytes were evaluated. 

## Materials and Methods

In this experimental study, unless otherwise specified, all chemicals and media were obtained from Sigma-Aldrich (St. Louis, Mo, USA) and Gibco (Grand Island, NY, USA), respectively. Similarly, all plastic dishes and tubes were obtained from Nunc (Roskilde, Denmark).

### Oocyte collection and *in vitro* maturation

Abattoir ovaries were obtained from goat and transferred in physiological saline at 35°C within 4 hours after collection. Cumulus oocyte complexes (COCs) were aspirated from follicles with 2-6 mm diameters. The procedure of in vitro oocyte maturation was performed as described previously (22). Briefly, selected COCs with more than two layers of cumulus were washed three times in HEPES-buffered tissue culture medium-199 (HTCM- 199) containing 10% fetal bovine serum (FBS). After washing, COCs (n=10) were transferred into 50 µL micro- drops of HTCM-199 supplemented with 10% FBS, 1µg/mL 17ß-estradiol, 5.0 µg/mL luteinizing hormone (LH), 0.5 µg/mL follicle-stimulating hormone (FSH), 100 IU/mL penicillin, 100 µg/mL streptomycin and melatonin under mineral oil. All cultures were incubated in maximum humidity with 5% CO_2_ at 38.5°C for 24 hours. 

### Experimental design

After 24 hours of culturing the cells, treatment groups with 10^-6^ M melatonin (M-10^-6^), 10^-9^ M melatonin (M-10^-9^) and 10^-12^ M melatonin (M-10^-12^), and the control group (without melatonin) were used in the designed experiments. The following were analyzed for each treatment and control group: nuclear maturation, GSH content, ROS levels, global DNA methylation, gene expression and developmental competence after parthenogenetic activation (PA). For each condition three to five replicates were used.

### Evaluation of nuclear maturation rate

For evaluating the transition from germinal vesicle (GV) to metaphase II (M-II) stage, COCs were striped from the cumulus cells mechanically in the presence of hyaloronidase and were fixed in 4% paraformaldehyde. Then, oocytes were washed in phosphate buffered saline (PBS) and stained with 5 µg/mL bisbenzimide (Hoechst 33342, excitation: 346 emission: 460) for 5 minutes. Stained oocytes were evaluated using epifluorescence microscope (Nikon Eclipse-600) for first polar body extrusion (23).

### Assessment of glutathione concentration

Glutathione content of oocytes was measured as described previously (24). Briefly, denuded oocytes were incubated in tyrodes medium (TLH) containing 5 mg/mL polyvinylalcohol (PVA) and 10µM CellTracker Blue (excitation: 371 emission: 464) for 30 minutes at 38.5°C. After incubation, oocytes were washed in PBS and observed using epi-fluorescence microscope (Nikon clips-300). Digital images were captured and analyzed by Image J software. 

### Analysis of reactive oxygen species level in maturation medium

The ROS production in centrifuged culture medium following IVM was measured by the chemiluminescence. One microliter of luminal (50 mM) dissolved in dimethyl sulfoxide was added to 400 µl of the supernatant. The global ROS levels were evaluated by measuring chemiluminescence with a luminometer (LKB 953, Wallac, Gaithersburg, MD) for 15 minutes, and the results were expressed in relative light units (RLU)/s (25, 26).

### Parthenogenetic activation and embryo development

Parthenogenetic activation method was described earlier (22). Briefly, after the maturation period, oocytes were stripped from cumulus cells by vortexing. Denuded oocytes were exposed to 5 mM inomycine for 5 minutes in HTCM and then washed three times in Charles Rosenkrans 1 with amino acid (CR1aa) medium. Afterward, oocytes were incubated for 2 hours in CR1aa medium contain 2 mM 6-dimethylaminopurine. Finally, activated oocytes (n=6) were transferred to 20 µl droplets of CR1aa plus 3 mg/mL bovine serum albumin (BSA) under mineral oil at 38.5°C, 5% O_2_, 5% CO_2_ and maximum humidify for 3 days, and then the medium was refreshed with 10% FBS. The cleavage and blastocyst rate were determined on day 3 and 8 post activation, respectively.

### Immunostaining of 5-methylcytosine 

After fixation in 4% paraformaldehyde, the oocytes were permeabilised with 1% Triton X-100 in PBS for 1 hour, then washed in Tween-20 in 1% PBS/BSA and treated with 2 N HCl for 1 hour at room temperature. After washing in Tween-20 in PBS, the samples were blocked in 0.5% Triton X-100 in 1% PBS/BSA for 1 hour. After blocking, the oocytes were incubated with primary anti-5-methyl cytosine antibody (mouse monoclonal, Abcam, Cambridge, UK) at 1:200 in the blocking buffer for 1 hour at room temprature. After incubation with the primary antibody, the samples were washed in PBS/BSA and incubated with phycoerythrin- conjugated secondary antibody (Molecular Probes, Invitrogen, Carlsbad, CA, USA). After the final wash in PBS/BSA, the DNA of oocytes was stained with 1 µg/mL Hoechst 33342 for 15 minutes. Oocytes were mounted on slides and observed with a Nikon (Eclips-300) fluorescence microscope and the fluorescence intensity of the oocytes was analyzed by Image J software (27).

### RNA isolation and reverse transcriptase-polymerase chain reaction

For each group, three pools of biological replicates containing (n=10) mature oocytes and their surrounding cumulus cells were used for total RNA isolation. RNA pellets were dissolved in sterile water and cDNA was synthesized using M-MULVE Reverse transcriptase. Briefly, 2 µg total RNA was mixed with 5 mM Random Hexamer. Five µL water was added to 2 µL of oocytes and incubated at 75ºC for 5 minutes for the reaction to take occur. Then 10 µL RT buffer, 10 mM dNTPs, 10 µL RNase inhibitor and 200 U reverse transcriptase were added to reach a total volume of 20 µL. Reverse transcriptase-polymerase chain reaction (RT-PCR) was done in an applied Bio Rad thermocycler. After the reverse transcriptase reaction was finished, the samples were maintained at 4ºC overnight. PCR reaction was performed in total volumes of 26 µL that included 2 µL cDNA, 2 µL of each primer and 1.25 µL tag polymerase, 20.75 µL Master Mix (Takara, Japan). The PCR primers for each gene are listed in Table 1. The endogenous control (YWHAZ) and the three investigated genes were amplified with PCR cycle program at 94ºC for 3 minutes followed by 40 cycles of 94ºC for 30 seconds and 72ºC for 45 seconds. The number of cycles varied between 30 and 40, depending on the abundance of a particular mRNA. Ten microliters of PCR product were mixed with 1 mL loading buffer and electrophoreses was carried out on a 2% agarose gel in TAE for 25 minutes. The ovary was used as a positive control for melatonin receptors (28).

### Quantitative real time-polymerase chain reaction analysis

Real-time quantitative RT-PCR was performed to assess the expression of the investigated genes by using Rotor Gene Q instrument (QIAGEN, Germany). Real time PCR reactions were carried out in a total volume of 13 µL according to the manufacturer’s manuals for DNA Master SYBR Green I Mix (Takara, Japan). The primer concentrations were adjusted to 1 µM for each gene. The cycling parameters were 5 seconds at 95ºC, 3 minutes at 95ºC for denaturation, 15 seconds at 60ºC, 10 seconds at 72ºC for amplification and 40 cycles of extension. Expression of YWHAZ transcript was used as the internal housekeeping gene. Three replications were performed and the mRNA level of each sample was normalized to that of YWHAZ mRNA level. The relative levels of mRNA were analyzed by the REST software (Qiagen, Germany) (6).

### Statistical analysis

The nuclear maturation of oocytes, cleavage and blastocyst rates were compared by x^2^ analysis. The intracellular GSH content and ROS levels were analyzed by one-way ANOVA followed by Tukey’s test via SPSS 22 for windows (SPSS, Chicago, IL, USA). Relative gene expression levels of different genes were evaluated by REST software. A P<0.05 was considered statistically significant. The data are expressed as mean ± SD.

## Results

### Nuclear maturation of goat oocytes

The effect of melatonin on nuclear maturation of goat oocytes was examined. Supplementing the IVM medium with melatonin significantly increased the rate of M-II oocytes at M-10-12 group (88%) when compared to the control group (76.1%) (Table 2). No significant difference (P>0.05) was observed between the other groups.

**Table 1:** Primer sequences used for gene expression


Gene name	Primer sequence (5ˊ-3ˊ)	Annealing temperature(c)	Product size (bp)	Accession number

MTNR1A	F: TCGCCTCCATCCTC	60	106	XM _005698759.1
	R: AACACATTCCCTGCGT			
DNMT3b	F: GAAGATCCTACAAAGACAG	60	115	NM_18181302
	R: AATTTTCCCCTCCTTCTCCTGC			
DNMT1	F: CGGAACTTCGTCTCCTTC	60	114	XM_015471996.1
	R: CACGCCGTACTGACCAG			
DNMT3a	F: AGCACAA CGGAGAAGCC	60	192	NM_001206502
	R: TTCCAGGAAGCAGTTCTTG			
YWHAZ	F: ATCTTGT GTCGTGTGGGG	60	140	XM_005689196.2
	R: CTCGG AGAACTTGCCATC			


**Table 2:** The effect of melatonin treatment on nuclear maturation in goat oocytes


Group	Number of COC’s	n (MII %)	P value	Odd ratio

Control	67	51 (76.1)	-	-
M-10^-6^	64	54 (84.3)	0.27	1.16
M-10^-9^	75	65 (86.6)	0.14	1.12
M-10^-12^	92	81 (88)	0.04^*^	1.23


*; Significant difference and COC; Cumulus oocyte complexes.

### Glutathione level in goat oocyte

**Fig.1:**
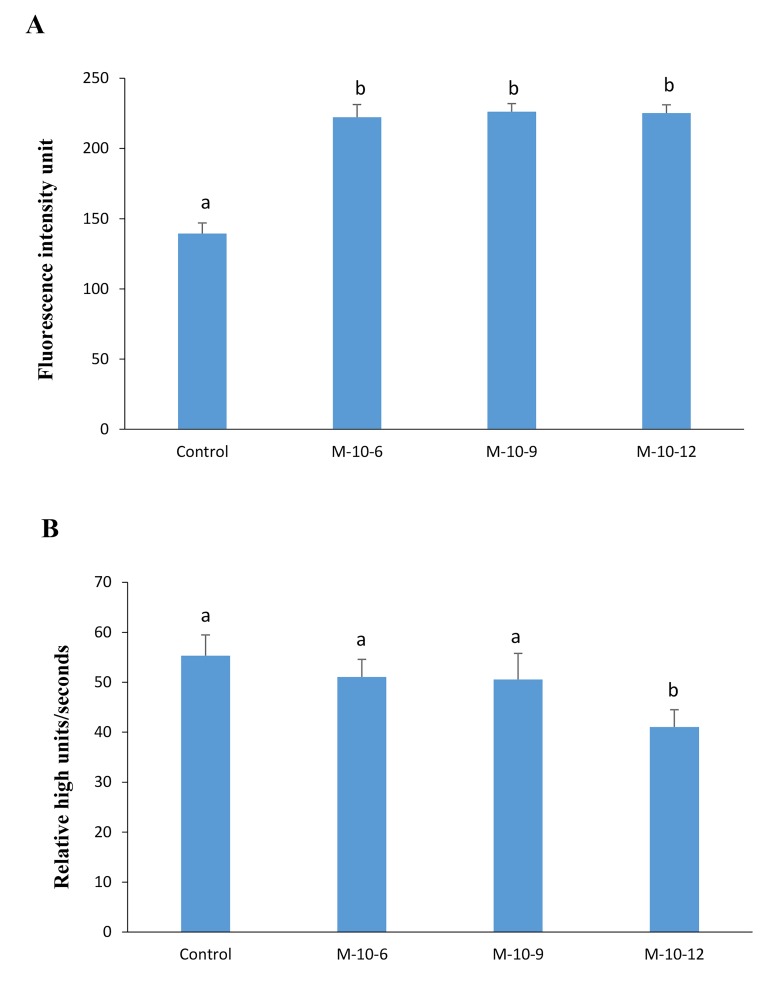
Glutathione (GSH) and reactive oxygen species (ROS) levels. The effect of melatonin on intracellular A. GSH and B. ROS levels in goat oocytes after in vitro maturation. Different letters (a, b) indicate a significant difference (P<0.05).

The obtained results from fluorescence intensity experiment (Fig .1A) indicate that GSH content was significantly higher in melatonin-treated groups compared to the control (P<0.05).

### Oocyte developmental competence

Our findings demonstrated that different concentrations of melatonin (i.e. M-10-6, M-10-9 and M-10-12) had no effect on cleavage rate after PA when compared to the control group. However, the blastocyst formation were higher (P<0.05) in M-10^-6^ (55.4%), M-10^-9^ (49.2%) and M-10^-12^ (51%) groups as compared to control group (34.7%). Moreover, in terms of blastocyst formation, no difference (P>0.05) was observed among melatonin-treated groups (Table 3).

### Effect of melatonin on the reactive oxygen species level of maturation medium

The present data indicate that M-10-12 group had a significant effect on ROS levels in comparison to the control groups (Fig .1B).

**Table 3:** The effect of different concentrations of melatonin during in vitro maturation on cleavage and blastocyst rates of goat oocytes after parthenogenesis activation


Group	Number of COC’s	Cleavage rate n (%)	P value	Blastocyst rate n (%)	P value

Control	92	72 (78)	-	25 (34.7)	-
M-10^-6^	95	74 (77.8)	0.99	41 (55.4)	0.01^*^
M-10^-9^	89	65 (73)	0.55	32 (49.2)	0.05^*^
M-10^-12^	66	45 (68.1)	0.13	23 (51)	0.04^*^


*; Significant difference and COC; Cumulus oocyte complexes.

### Changes in DNA methylation in goat oocytes treated with melatonin

**Fig.2:**
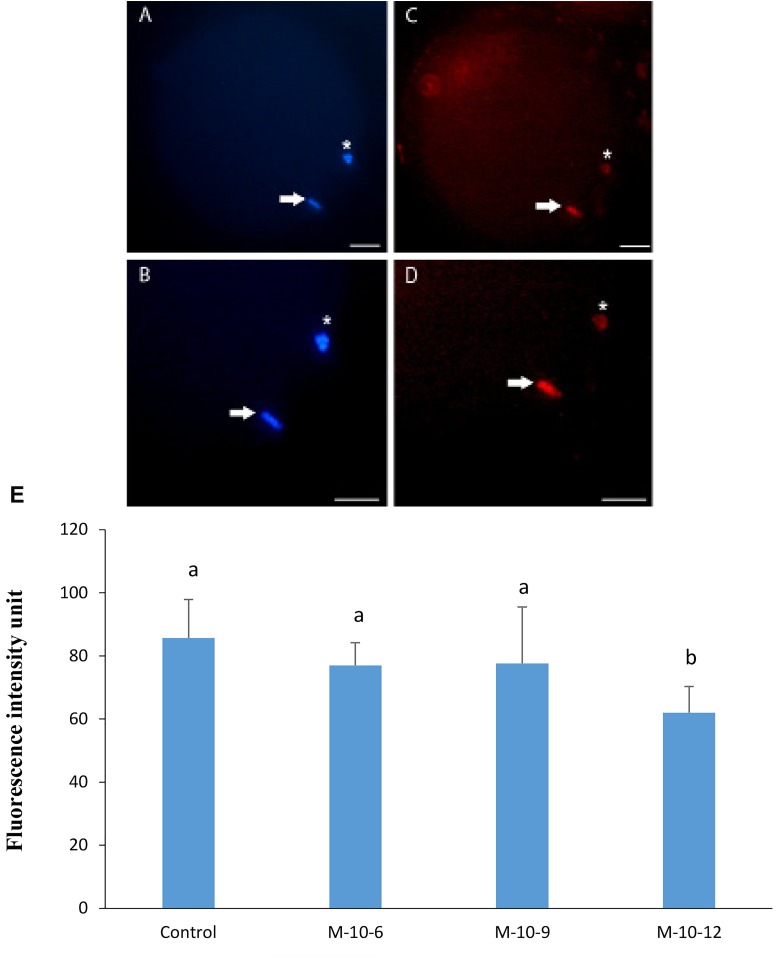
Immunocytochemical staining of oocyte. Oocytes stained with A, B. Hoechst followed by C, D. Methyl cytosin labeling in goat oocytes. *; Indicated polar body and arrow indicated M-II plate (scale bar: 20 µm), and E. Changes in methylation levels in goat oocytes from the experimental and control groups, as estimated by immunostaining. Different letters (a, b) indicate a significant difference (P<0.05).

Representative images of labeling for 5-methyl cytosine in goat oocytes are shown in Figure 2A-D. Results from quantitative analysis of these images by Image J software showed significantly different methylation levels between the control and M-1012 groups (Fig .2E).

### The effect of melatonin on the expression of DNA methyltransferase genes

**Fig.3:**
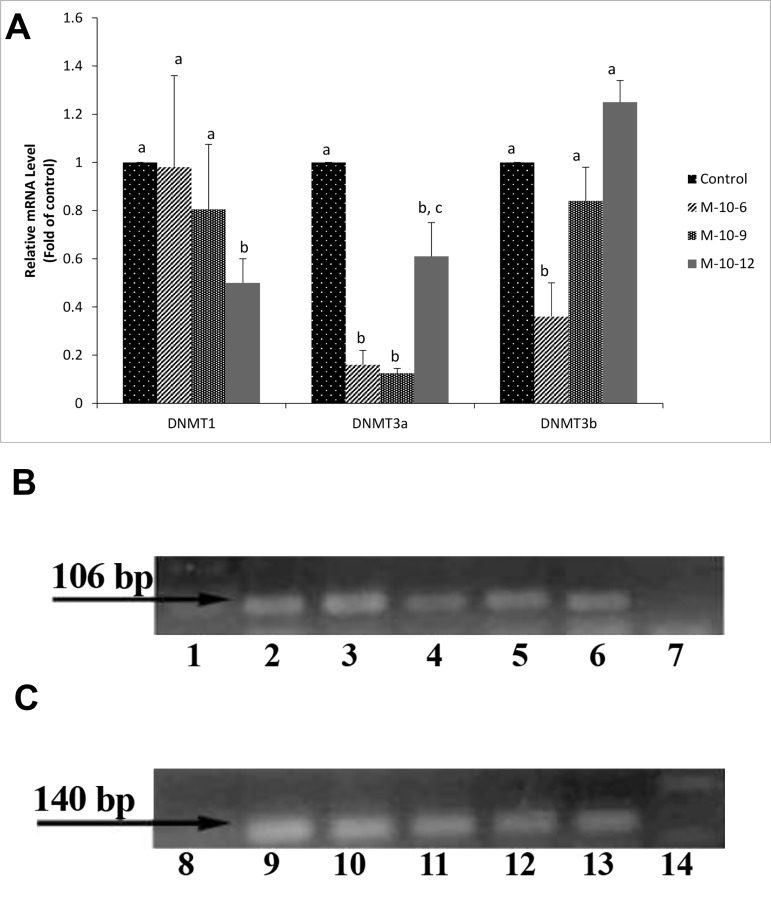
Gene expression following oocyte *in vitro* maturation. A. The expression of DNMTs genes in goat matured oocytes treated and un-treated with melatonin. Different letters in each gene group indicate significant difference in gene expression, B. The expression of melatonin receptor (*MTNR1A*) in mature goat oocytes treated and un-treated with melatonin (lanes 2 and 3) and cumulus cells from matured oocyte treated and un- treated (lanes 4 and 5). Lane 1 shows the DNA molecular weight marker (100 bp ladder). Lane 7 shows the polymerase chain reaction (PCR) reaction without cDNA substrate as the negative control, and C. The expression of *YWHAZ* in goat matured oocytes treated and un-treated with melatonin (lanes 10 and 11) and cumulus cells from matured oocyte treated and un-treated (lanes 12 and 13). Lane 8 shows the DNA molecular weight marker (100 bp ladder). Lane 14 shows the PCR reaction without cDNA substrate. Lane 6 and 9 in both pictures show the expression of *MTNR1A* and *YWHAZ* in ovary tissue as a positive control.

The expression of DNMTs genes were analyzed by quantitative real-time PCR in mature oocytes (Fig .3A). The expression of *DNMT1* in M-10^-12^ group was significantly lower (P<0.05) in comparison to the control group. Our observations indicated that the expression of *DNMT3a* was lower significantly in all melatonin-treated groups compared to the control group. The expression of *DNMT3b* was significantly lowered in the oocytes with melatonin 10^-6^ treatment compared to the control groups (P<0.05).

### The effect of exogenous melatonin on the expression of melatonin receptor

The expression of *MTNR1A* gene was detected via RT- PCR in both oocytes and cumulus cells, in response to melatonin addition to the IVM medium (Fig .3B, C). This result shows that *MTNR1A* exists in both oocytes and cumulus cells independent from the presence of melatonin in the maturation medium.

## Discussion

A considerable amount of studies on melatonin indicates that it is a multifunctional antioxidant molecule, mediating several circadian and seasonal reproductive processes, as well as acting as a radical scavenger (29, 30). Therefore, in this study we investigated the effects of melatonin on oocyte maturation and embryo development in goats, which are important farm animals. Also, the expression of *MTNR1A* and DNMT-related genes in goat oocytes were analyzed.

Our results indicated that melatonin at 10^-12^ M has a significant effect on first polar body extrusion. These results are consistent with previous findings, in which melatonin was shown to be an essential factor for first polar body extrusion in porcine, bovine and mouse (15, 20, 31). 

In some studies, GSH level in oocytes is used to evaluate cytoplasmic maturation of the oocytes; in fact, GSH is known to be an important intra-oocyte factor for developmental competence (32). Data from bovine and mouse shows that GSH level in embryos treated with melatonin increases significantly after IVM and vitrification (6, 31), which is consistent with our results.

In this study, we used parthenogenesis technique, because in this method developmental competence of the oocytes is completely independent from sperm effects. Our results indicated that after activation, melatonin increases blastocyst rate but does not have any significant effects on cleavage rate. These data are consistent with data from porcine (14), bovine and mice, (6, 33), but in contrast with results from ovine (34). This discrepancy may be due to the species specific effect of melatonin or technical factors that can influence developmental competence of the oocytes (35, 36).

According to our results from GSH and blastocyst formation, we can argue that supplementation of melatonin in the maturation medium improves cytoplasmic maturation of the oocytes, which has a beneficial effect on developmental competence of the oocytes following parthenogenesis. Therefore, melatonin, like other antioxidants including resveratrol, can be used in maturation medium and protect oocytes from harmful effects of ROS (35).

This study revealed that melatonin treatment during goat oocyte maturation decreases the expression level of *DNMT1* and *DNMT3a*, which have vital roles in increasing transcription and expression of other genes (36). In addition, our results from immunofluorescence assay indicated that melatonin lowers global methylation level in goat oocytes.

It has been clearly established that *in vitro* production of an embryo has adverse effects on DNA methylation (37). Other studies have shown that porcine embryo, which was produced in vitro, has higher levels of DNA methylation in comparison to those produced *in vivo*. For this reason, researcher have used drugs for manipulating epigenetic outcomes after nuclear transfer. However, some of them were toxic and their usage requires further experiments. For example, 5-Aza-2-deoxycytidine had an effect on DNA hypomethylation with no effect on H3K9 hyperacetylation (38). Recent have indicated that melatonin, as a natural antioxidant, can be used in cancer research, similar to procaine and hydralazine, which are known as methyltransferase inhibitors (39). So, based on our results melatonin can be used in IVM for regulation of DNA methylation levels. 

In this work, we also examined the expression of *MTNR1A* in oocytes and cumulus cells. Our results confirmed the expression of *MTNR1A* in both cumulus and oocytes in goat model by using RT-PCR. This is an important aspect of our report, as it is presenting data on a different kind of reproduction-regulating receptor compared to previous studies (31). 

## Conclusion

Supplementation of melatonin at different concentrations during IVM of oocytes improved the potential development of parthenogenetic embryos. This improvement is due to increased amount of intracellular GSH, decreased ROS levels and decreased abundance of DNMTs gene transcripts in mature oocytes, which are all important in nuclear methylation and gene expression. In addition, *MTNR1A* expression was detected in both cumulus cells and oocytes of the goat.
